# Stimulant Usage by Medical Students for Cognitive Enhancement: A Systematic Review

**DOI:** 10.7759/cureus.15163

**Published:** 2021-05-22

**Authors:** Noorine Plumber, Maliha Majeed, Shawn Ziff, Sneha E Thomas, Srinivasa Rao Bolla, Vasavi Rakesh Gorantla

**Affiliations:** 1 Department of Anatomical Sciences, St. George’s University School of Medicine, Grenada, GRD; 2 Department of Internal Medicine, University of Maryland Medical Center, Baltimore, USA; 3 Department of Biomedical Sciences, Nazarbayev University School of Medicine, Nur Sultan, KAZ

**Keywords:** energy drink, smart pills, stimulant, cognitive enhancer, amphetamine, caffeine

## Abstract

Stimulants have been used throughout human history for a variety of reasons. High levels of stress and the demanding nature of medical school make their usage among medical students particularly common. The most prevalent stimulant used by students is coffee, followed by tea and other forms of caffeine like sugary energy drinks. In addition, amphetamine-based medications for treating attention deficit hyperactivity disorder (ADHD) have been increasing in popularity, which many students take illicitly. Students report taking various forms of stimulants to promote cognitive enhancement, prolong wakefulness and retain focus for long periods of time. Moderate doses of caffeine and amphetamines would lead to enhanced alertness and concentration. However, large increases in dosage or frequency would lead to an increased risk of toxicity and adverse effects. The positive outcomes from stimulant consumption are often overshadowed by the negative side effects and incorrect dosage. Thus, it appears that usage of stimulants should be limited, in favor of a more sustainable approach to cognitive enhancement.

This review analyzes the use of stimulants among the medical student community, consequences of misuse and discussed the healthy and organic approaches to lessen the stress and improve academic performance. This article also discusses the mechanisms of action, acceptable doses, additives, ingredients of stimulants commonly used by medical students for cognitive enhancement and the implications of long-term use as the stress of practicing medicine extends well beyond the medical school years.

## Introduction and background

The use of stimulants in a variety of forms is common practice among a majority of the world’s population. The popularity of misuse likely stems from the idea that the use of these stimulants will improve focus, attention, and thus, academic performance. As a matter of fact, most medical students who reported the use of stimulants started taking them during their first year and were seeking out alertness and wakefulness for competitive exams [1]. Students may struggle to perform well on early-morning exams and therefore rely on stimulants that make them alert in hopes of higher scores. Some studies discuss that the use of stimulants may provide an unfair advantage in the form of increased attentiveness or improvement in academics [2]. It is possible that those who take the stimulants are struggling academically and the focus enhancement from the drug leads to their grade point average (GPA) being average when compared to those who do not rely on stimulants. Although, studies show that while these drugs increase focus, those that use amphetamines do not have a higher GPA than non-users [2]. Studies also suggest that excessive stimulant use results in decreased sleep quality and quantity, which can lead to increased fatigue [3]. Stimulant users may perceive this fatigue as an indication of requiring even more stimulants, however, this requires further research and evaluation.

The fluctuations in usage, overuse, and in some cases misuse of these stimulants have led to the recommendation that stimulant usage should be limited and the consumer should be more aware of the effects these compounds have. In order to address these concerns, this systematic review discusses how students and the institutions they are a part of should approach the topic and what actions should be implemented to ensure healthy practices. Through the analysis of existing literature, this paper aims to further the discussion of stimulant use among medical students, their mechanism of action, whether or not the intended outcomes are achieved, and the presence of side effects.

## Review

**Figure 1:**
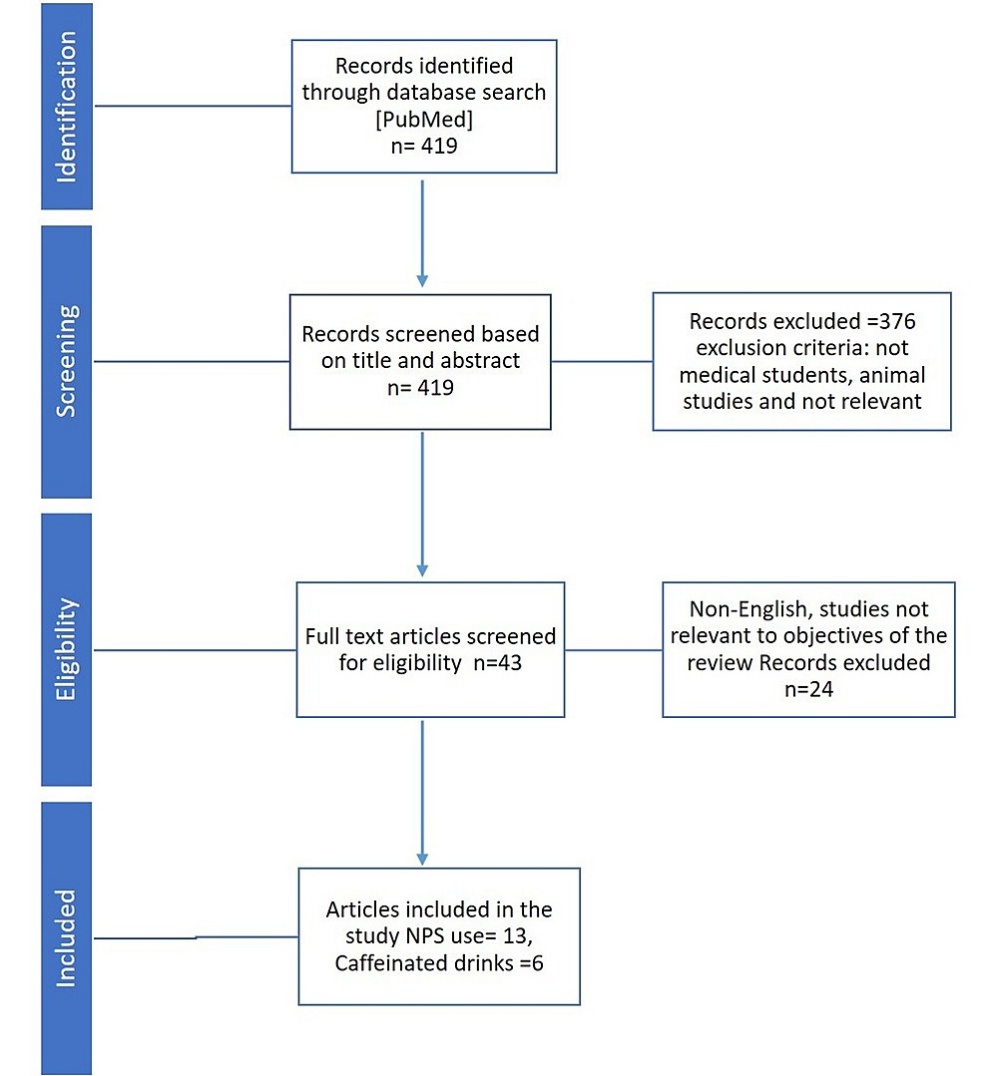
PRISMA flowchart of the literature screening for NPS use among medical students. Screening of the literature has been done as described in the PRISMA statement [4]. PRISMA: Preferred Reporting Items for Systematic Review and Meta-Analyses; NPS: Nonprescription stimulant.

A search of the literature was done in the PubMed database for articles from 2010-2021 with the keywords (caffeine OR amphetamine OR energy drink OR smart pills OR stimulant OR cognitive enhancer) AND medical students on 29th April 2021. Non-English articles not relevant to medical students, and articles not relevant to the study objectives were not included. Studies relevant to the use of caffeine and nonprescription stimulant (NPS) use among medical students to enhance academic performance or to cope with academic stress were included (Figure 1).

Results

Our search yielded 419 results of which 13 articles [1,5-16] were included for NPS use and six articles [17-22] for high caffeinated drinks use. The studies on NPS use among medical students totaled 11029 medical students and 970 among them were using NPS [1, 5-16]. There were six studies on caffeinated drink use among medical students (Table 1) which included 3154 students, and among them most used coffee as a source of caffeine and some of them used high caffeinated energy drinks to cope with academic stress and wakefulness [17-22].

**Table 1:** Studies on the use of stimulants among medical students Table showing the studies on the use of stimulants for cognitive enhancement from 2010-2021. It includes the nonprescription stimulants (NPS) used for attention deficit hyperactivity disorder (ADHD) treatments and caffeinated drinks.

Non-Prescribed Stimulant use						
	Author	Country	Intervention	Study population	Findings	Conclusion
1	Miranda and Barbosa, 2021 [5]	Portugal	Use of cognitive enhancers	1156 medical students (913) and licensing exam applicants (243)	48 (5.2%) medical students, 35 (14.4%) licensing exam applicants were using NPS. Methylphenidate and modafinil were used by most. Coffee and energy drinks were used by others.	Public health, ethical and medical concerns of NPS use among medical students
2	Alrakaf et al., 2020 [6]	Saudi Arabia	Prevalence of NPS use	1,177 medical students	68 (5.8%) participants used ADHD drugs, of these 39 (3.31%) were prescribed and 29 (2.46%) used them illicitly. Adderall, and Ritalin (methylphenidate).	Students need to be educated and provide healthy stress coping methods
3	Acosta et al., 2019 [7]	Puerto Rico	Prevalence of NPS use of ADHD medication	152 medical students	47.4% (72) used ADHD medication and 86.8% used coffee, energy drink, etc. to cope with academic stress	Non-medical use is a public health concern, stress coping workshops to help medical students are to be incorporated
4	Haas et al., 2019 [8]	Brazil	NPS use among medical students	698 medical students	63 used ADHD medication without a prescription, motivation is to study longer and increase concentration. methylphenidate or lisdexamfetamine was used	Devise plans to curb stimulant use
5	De Bruyn et al., 2019 [9]	Belgium	NPS use among medical students	3159 medical students	237 used NPS during exams. methylphenidate, modafinil, and amphetamine.	Underlying causes for NPS use need to be explored and addressed
6	Fallah et al., 2018 [10]	Iran	Stimulant use among medical students	260 medical students	49 (11%) used NPS. Ritalin, amphetamine	Promote life skills, awareness of side effects of the NPS early in the school are proposed
7	Retief and Verster, 2016 [11]	South Africa	Prevalent of NPS use and correlations	252 fourth medical students	42 (17%) used NPS. ADHD drugs	NPS use is prevalent among medical students for improving concentration. Further study needed to find prevalence in other schools.
8	Fond et al., 2016 [1]	France	Estimate the prevalence of NPS	1718 medical students and physicians	499 (29.7%) used caffeine tablets and/or energy drinks containing high dosage of caffeine, 113 used NPS	The study reported a high rate of students using stimulants
9	Wasserman et al., 2014 [12]	USA	Prevalence of NPS use among Osteopath students	380 medical students	56 (15.2%) used NPS. ADHD medication	Did not find a correlation between NPS use and academic stress. Suggested to naturally engage students in the academic environment.
10	Kudlow et al., 2013 [13]	Canada	Evaluation of cognitive enhancement NPS use in medical students	326 medical students	49 (15%) used NPS. methylphenidate (Ritalin) (24 [7%]), modafinil (Provigil) (18 [6%]), dextroamphetamine (Dexedrine) (11 [3%]), dextro/levoamphetamine (Adderall) (10 [3%]), adrafinil (Olmifon) (5 [2%]), and piracetam (5 [2%]). 117 used high caffeine products	Considerable student population used NPS. Usage increased in upper years of medicine program
11	Emanuel et al., 2013 [14]	USA	To find NPS use among medical students	1115 medical students	117 (11%) used NPS during medical school. amphetamines or methylphenidate	The study provided data on the prevalence of psychostimulant use in medical students
12	Habibzadeh et al., 2011 [15]	Iran	To find the frequency of methylphenidate use in medical students	310 medical students	27 (8.7%) had taken methylphenidate, three of them used by physician’s prescription	There is less awareness about methylphenidate use.
13	Tuttle et al., 2010 [16]	USA	To find the prevalence of ADHD and NPS use among medical students.	326 medical students	33 (10%) students use NPS	Medical students are high-risk people for NPS use
Caffeinated Drinks						
1	Samaha et al. 2020 [17]	Lebanon	Stress and caffeine addiction	596 medical students	Caffeine source was coffee in 528, energy drinks in 209, some used both	Data on trends of caffeine use in medical students
2	Aslam et al., 2013 [18]	Pakistan	Evaluation of consumption and awareness of energy drinks	866 medical students	350 (42.89%) were using energy drinks	Students reasoned exam stress and long waking hours for Energy drinks. Need for awareness of side effects is recommended.
3	Hidiroglu et al., 2013 [19]	Pakistan	Finding energy drink consuming pattern	390 medical students	Students consumed Energy drinks once were 127 (32.6%), more than once were 73 (18.8%).	Though students were consuming Energy drinks, they lack knowledge about ingredients and side effects.
4	Ríos et al., 2013 [20]	Puerto Rico	To find an association between caffeinated drinks and academic load, stress	275 medical students	88% consumed caffeinated drinks	Caffeinated drinks are popular but there is no association between their consumption and academic load.
5	Casuccio et al., 2015 [21]	Italy	Evaluate knowledge, attitudes, and practices of energy drink consumption	794 medical students responded	173 regular energy drink consumers (22%). 77 of them reported side effects (palpitations, insomnia, irritability, anxiety, etc.)	Large usage of energy drinks with or without alcohol was found. Future work is needed to assess long-term and short-term side effects.
6	Usman et al., 2015 [22]	Pakistan	Estimate prevalence of energy drink consumption	233 medical students	121 (51%) reported consuming energy drinks	Found high prevalence of energy drink consumption. Programs on awareness about the energy drinks are to be done

Discussion

As stimulant use, namely caffeine and amphetamine becomes increasingly prevalent, this paper sought to explore the use of stimulants among medical students given the above-average amount of stress in their daily lives. Increased use of these stimulants can be correlated with communities in which there are high amounts of stress such as prior to an exam [6]. This includes students, who are met with the burden of a large workload and the pressure to do well. Highly specialized postgraduate programs like medical training are competitive and have demanding environments and as a result, many students have incorporated stimulatory agents use into their routine. Students report using these drugs for a variety of reasons, often citing academic performance as a motivating factor [1]. The most prevalent stimulant used by students is coffee, followed by tea and other forms of caffeine-like sugary energy drinks. In addition, the use of nonprescription stimulants such as amphetamine salt-based ADHD drugs is increasing. Students are also more likely to turn to highly caffeinated energy drinks in such instances as they contain a surplus of caffeine and promise to energize the consumer.

Studies show that excessive and unregulated consumption of these drinks results in deleterious and adverse physiological effects that are often prolonged and worsened due to lack of education and awareness. The increased utilization of energy drinks is associated with an increased likelihood that the student will consume substances such as alcohol or drugs that are marketed to promise cognitive enhancement [17, 20].

Nonprescription stimulant among the medical student community

In recent years, there has been a steady rise in popularity in the use of schedule 2 drugs among students for cognitive enhancement [23]. Amphetamine salts are widely indicated for the treatment of attention deficit hyperactivity disorder (ADHD). Despite efforts to regulate the drug secondary to its potential for addiction and abuse [24], the ease of attainment has led to an increased frequency of use, specifically among students.

From the review of literature, we found these stimulants are often consumed to increase focus for academic purposes but may provide a feedback mechanism through emotional centers that lead to unwanted outcomes. Taking these results into consideration, a recommendation for future research would be to look for possible placebo effects with the use of Adderall in healthy brains as students using them for cognitive enhancement might feel motivated because simply taking a substance implied to improve focus. Studies show that the population is not educated on the mechanism of action, side effects, and implications of the use of psychostimulants such as amphetamines for academic performance [6]. The most consumed NPS are ADHD medications such as Adderall (mixed salts amphetamine), dextroamphetamine, and Ritalin (methylphenidate). The use of the NPS ranged from 5.2% to as high as 47.4% among the medical students across different countries (Table 1). Jain et al. questioned medical students in their basic science years on their knowledge of the effects of these drugs. Results indicated that although knowledge increased as students progressed in their coursework, overall knowledge was low. Those who used the drug were slightly more knowledgeable on its side effects [25].

Although amphetamines, namely Adderall, are a widely accepted medication, the illicit use of it in the medical student community for performance enhancement should be evaluated. It is recommended that students be educated on the drug as part of their curriculum and students ought to be cautioned considering that the use of regulated drugs without a prescription is illegal and selling them to other students is a criminal offense [14]. However, addressing the use of NPS, its effects on neurobiological processes, dependence, and possible negative outcomes is required. High-stress levels are noted in medical schools and extend into residency and the career of a physician. The addictive properties of stimulatory agents can lead to reliance on those that start using them to deal with stressful circumstances of school [14]. The ease of accessibility, whether from peers, acquaintances, or the internet, profoundly increases the risk of usage. This is partly due to the fact that when obtained in this manner the regulatory process is circumvented, compared to receiving prescription medication through a pharmacy [14].

Amphetamine - Mechanism of action and side effects

Medications such as Adderall, prescribed to patients mainly to treat ADHD, have been used by students in an attempt to boost academic performance, for recreation, and to maintain wakefulness over long periods of time [26]. The mechanism of action is important in order to understand the extent of its effect on the body. The active agents in Adderall are made of a combination of amphetamine salts, thus we will focus on the effect of amphetamines. When students use Adderall or other ADHD medications the vast majority do so by ingesting a pill or capsule orally. Amphetamines work on both the central and peripheral nervous systems and because they are lipid-soluble, they are able to rapidly cross the blood-brain barrier [27]. Amphetamines work primarily on the brain by altering the amount of dopamine and norepinephrine available to post-synaptic neurons. By inhibiting the uptake transporters of both dopamine and norepinephrine, they are able to increase the amount of dopamine and norepinephrine within the synaptic cleft [28]. However, amphetamines have a similar mechanism of action on serotonin, epinephrine, and histamine to varying degrees [27]. Students who engage in the use of stimulants often do so without fully understanding the effects. Weyandt et al. found that the use of Adderall led to significantly reduced variability as well as marginally reduced Commission Errors and Hit Reaction Time, considered to be a marker of cognitive enhancement. They also found Adderall worsened working memory. However, the most significant changes were recorded in the test subject’s emotional response [29].

Amphetamines are taken up by the presynaptic neuron through a channel protein known as dopamine active transporter (DAT). The amphetamine molecule then does one of two things, it activates Trace-amine associated receptor 1 (TAAR1) (an intracellular G-protein coupled receptor) or it enters the vesicles which house the presynaptic dopamine molecules through a transporter known as vesicular monoamine transporter 2 (VMAT2) [30]. Upon entering the vesicles, the amphetamine molecule causes dopamine to be released into the cytosol. Amphetamine has also been observed to inhibit the activity of MAO (monoamine oxidase) which would normally break down the cytosolic dopamine, leading to further increased availability of the neurotransmitters [29]. Additionally, the binding to TAAR1 causes activation of protein kinase A (PKA) and protein kinase C (PKC) to upregulate phosphorylation of DAT, decreasing its activity and causing it to withdraw from the membrane [30]. All of this has the result of increasing the amount of catecholamines present in the synaptic cleft, specifically dopamine and norepinephrine. The excess catecholamines present in the cleft propagates the neurotransmitter’s signal and causes the effect associated with amphetamines.

The increased propagation of dopamine has been observed in several parts of the brain. Studies using positron emission tomography (PET) and single photon emission computed tomography (SPECT) scans in healthy adults have shown that amphetamines lead to an increase in dopamine release in the dorsal and ventral striatum, substantia nigra, and regions of the cortex [28]. The cortex is an especially important area for executive control and function, such as planning, goal-directed behavior, inhibition, working memory, and the flexible adaptation to context [28]. Increased levels of dopamine in the brain have been linked to both euphoria as well as psychosis and the degree of the effect has been shown to be dose-dependent [27,28].

Negative side effects have been associated with the use of amphetamines, including weight loss, loss of appetite, and insomnia [28]. Amphetamine toxicity has been observed to interfere with the N-methyl-D-aspartate (NMDA) receptors, which may lead to seizures [27]. In addition, amphetamines have also been linked to increased blood pressure and heart rate, further increasing risk to individuals with underlying heart conditions [27]. Due to the effect that amphetamines have on neurotransmitters associated with the reward center, they may be susceptible to addiction and abuse.

Caffeine and energy drinks use

Almost 89% of the United States’ population and 80% of the world’s population consumes caffeine for its psychostimulatory effects. The multi-billion-dollar caffeine production and advertising industry only grow each year as it is the main and sought-after ingredient in energy drinks, soda, and chewing gum, chocolates, cosmetic products, and countless items. As a high-stress and mentally challenging field, there are correlations between the non-medical use of amphetamines and other stimulants and the engagement of the individuals who use them in high-risk behaviors [31]. This is also seen in students who drink energy drinks with high caffeine content [31]. While the use of energy drinks as a source of caffeine is minimal compared to tea and coffee, it was observed that the use of them was increased around high-stress times, like exams [12]. Many of the students consumed coffee and up to 50% of the samples studied are consuming energy drinks to cope with the stress (Table 1). Stress and long wakeful hours are reasoned for their consumption [12]. The danger in these behaviors stems from the idea that most people are unaware of the ingredients of energy drinks and drink them simply for a burst of energy [32]. The high sugar and caffeine contents of energy drinks mixed with ethanol minimize the depressant effects of alcohol and mask the signs of intoxication. This greatly increases the chances of accidents while intoxicated and increases the risk of alcohol dependence with continued exposures [33].

However, most students in medical school report the moderate use of coffee as a source of caffeine, with the amount only slightly increased with high-stress situations like exams [34]. The use of stimulants among students requires further study and discussion so that healthy individuals have adequate information on what constitutes safe use of stimulants and what is considered abuse/misuse. Furthermore, students should be encouraged to review their own practices and compare them to data collected on the performance-enhancing capabilities in healthy test subjects. Many students report a marked increase in activated emotion [29], this may lead to the student’s continued use of a drug regardless of whether it is providing them with an academic benefit. Other students may overestimate the drug’s cognitive enhancing ability partially due to their own misunderstanding of how the drug achieves its mechanism of action. It seems unlikely that stimulant use will decrease in the near future, however, through further research and discussion, we can help to educate students on proper use to promote a healthy and productive lifestyle.

Caffeine - Mechanism of action and side effects

Caffeine (1,3,7-trimethylxanthine) has become the most widely used psychostimulant in the world, as it is easily accessible in a variety of beverages, food items, and supplements. This makes caffeine an especially important topic to discuss and understand its derivation, mechanism of action, and some of its effects on human health. Caffeine is found in several plant species, forms naturally in cacao beans and is extracted for widescale commercial uses [35].

Studies show that caffeine’s desired effects of increased attention and alertness are achieved at lower doses (<4 g) and some of its negative effects are seen at higher levels of consumption [35]. Some of its desired effects in particular include increased concentration, wakefulness, and faster reaction times. Long-term effects of moderate regular doses of caffeine may even reduce the risk of dementia and cognitive decline [36]. These effects are often sought for by individuals in physically and mentally demanding careers. Caffeine’s less desired side effects at much higher doses such as more than 5 grams include tremors, anxiety, and palpitations, among many more adverse effects. Toxic doses of caffeine have resulted in profound palpitations, worsening anxiety, and life-threatening arrhythmias such as supraventricular tachycardia ventricular fibrillation that require immediate medical attention [35]. Sherman et al. compared the effects of caffeine at optimal and non-optimal times of the day. Several factors were examined such as memory and reaction times in University of Arizona undergraduate students. During the non-optimal time of day, the study found that students had improved explicit memory and wakefulness. However, caffeine intake did not show improvements when consumed in the afternoon hours compared to the control group of participants [37]. Often the average caffeine consumer is unaware of such dangerous side effects of high caffeine doses and may unknowingly consume toxic doses due to its prevalence in many food items.

Caffeine’s lipophilic properties allow for efficient absorption through the gastrointestinal tract and through the blood-brain barrier. Caffeine primarily acts as a phosphodiesterase inhibitor on adenosine receptors, which results in increased intracellular concentrations of the second messenger cyclic adenosine monophosphate (cAMP). It targets several types of adenosine receptors present on various organs such as the brain, cardiac muscle, kidneys, and vasculature throughout the body. The increased concentrations of cAMP potentiate its effects through the signal transduction pathways. In cardiac muscle, caffeine increases inotropy, which results in a stronger force of contraction and therefore increased stroke volumes. It also increases chronotropy, which can result in increased heart rates and palpitations at high doses [38].

Caffeine indirectly increases the release of catecholamines, causing increased vascular tone and vasoconstriction. In the gastrointestinal tract, caffeine potentiates the effects of gastric acid secretion and motility [38]. These effects can have an especially profound impact on individuals that consume high doses of caffeine as their regular routine. Additionally, individuals that have comorbidities such as hypertension, cardiovascular disease, anxiety, and gastrointestinal conditions may encounter worsening and varied effects of caffeine consumption. Caffeine also affects other signal transduction pathways through ryanodine receptors, GABA receptors along with the storage and secretion mechanism of calcium ions [38]. However, these effects can be further discussed and analyzed in pharmacokinetics and metabolism studies.

Social and lifestyle habits also play a significant role in the effects of caffeine consumption and metabolism. Studies show that smokers metabolize caffeine at higher rates and thereby, reduces its half-life by almost 50% [38]. Conversely, pregnant patients and neonates have significantly increased caffeine half-life of up to 15 hours. Patients with renal and hepatic comorbidities may have increased caffeine half-life as well due to impaired metabolism and excretion functions [38]. High doses of caffeine consumption and caffeine toxicity can result in adverse effects such as those mentioned previously, as well as fatal arrhythmias. Although caffeine may have stimulatory effects of alertness and increased concentrations, high doses may lead to dependence and even tolerance [39]. The tolerance of caffeine doses may cause students and those in high-stress environments or careers to consume even higher doses of caffeine in the form of coffee, energy drinks, or caffeinated pills.

Presence of additives in energy drinks and lack of education

The use of caffeine may be as mild as one cup in the morning to aid in alertness and wakefulness, however, high stress may lead to an increased intake throughout the day. One cup of coffee contains about 95 mg of caffeine. While some might augment this by increasing the number of cups, some people, particularly younger populations including students turn to energy drinks as an alternative to coffee. The most popular include Red Bull energy drinks which contain 110 mg of caffeine per can and Monster energy drinks which contain 160 mg of caffeine per can, other brands vary but contain similar quantities [40]. Increased consumption of caffeine in students at irregular times, particularly in the form of sugary energy drinks, is linked to exams. While such usage is often acute in nature, chronic high doses of caffeine alter the circadian rhythm, tolerance to glucose, as well as insulin response [41]. Thus, individuals chronically consuming energy drinks late in the day are shown to have decreased tolerance to the high doses of sugar, secondary to increased insulin resistance and decreased production of insulin in response to the glucose [40].

While there is an increased caffeine content in these drinks per can as compared to a cup of coffee, additional ingredients are often unknown or varied among brands. Frequently, high contents of sugar or artificial sweetener can be found in energy drinks to the point of equating them to soft drinks. With an increasing incidence of obesity throughout the world, particularly in the United States, this is of concern particularly as sugar consumption in excess of the World Health Organization (WHO) recommendation is widely common [42].

Popular ingredients include L-carnitine, taurine, and ginseng among others. Studies postulate that taurine possibly functions as an antiarrhythmic to negate the effects of excess caffeine [43]. Further, while additives of energy drinks attenuate some effects of caffeine, they amplify others. It has been found in animal studies that an effect of caffeine is the upregulation of cortisol secretion into plasma via activation of the hypothalamic-pituitary-adrenal system. This is further augmented in habitual energy drink consumers as taurine and its derivatives increase serum cortisol production and secretion. This was reversible as discontinuing consumption decreased adrenal zona fasciculata hyperplasia seen in chronic use [44].

Fletcher et al. found that compared to caffeine from other sources such as coffee, participants who consumed energy drinks had a significantly higher corrected QT interval and systolic BP two hours after consumption [43]. In terms of safety, the Food and Drug Administration (FDA) requires that all new drugs that affect the QT/QTc interval be comprehensively investigated, however, since the main drug in these energy drinks is caffeine, further investigation is not required [44]. While there is no evidence of significant consequences of short-term daily consumption of energy drinks over weeks, long-term daily use may possibly be correlated with cardiovascular problems such as arrhythmias. Further investigation is warranted [43].

While the literature on the negative effects of caffeine in conjunction with taurine is widespread, the neuroprotective role of caffeine may also be evident in taurine. The quantities of taurine present in the brain significantly decrease with age. However, animal experiments mimicking Alzheimer’s show decreased cognitive impairments in rats treated with 60 to 120 mg/kg/day of taurine [45]. While this sounds promising, this must be taken with a grain of salt as there is a delicate balance of taurine homeostasis evident in the fact that disturbances have been reported in conditions such as autism and epilepsy [44].

Non-pharmacological interventions to deal with stimulant use

A vast majority of medical school curriculums include sessions on health, wellness, professionalism, and ethics. Incorporating education on professionalism and coping mechanisms such as fitness and, relaxation is important. With the current opioid epidemic and the increasing data suggesting the wide usage, the need to address these topics and educate medical students during basic sciences years may be beneficial. As medical students continue their education and practice in this mentally and physically demanding career, they are continuously at risk for physical, social, and psychological burnout. Researchers performed a meta-analysis that compiled data from 167 cross-sectional studies and 16 longitudinal studies to understand the prevalence of depression and suicidal ideation among medical students. Researchers found that the prevalence of depression or depressive symptoms and suicidal ideation among medical students was 27.2% and 11.1%, respectively [32]. Medical schools recognize that students are functioning at increased stress levels and promote psychological awareness and even require students to engage in health and wellness classes [46].

Joice et al. researched the role of yoga in attention and memory among medical students. In this study, 100 healthy medical students were recruited, and participants that had practiced yoga in the past one year were excluded from the study. Students in the experimental group practiced yoga with certified yoga training over 12 weeks, 30 minutes a day, and five days a week. Attention and memory were assessed quantitatively through PGMI memory scores. Researchers found a significant increase in attention (p < 0.001; t = 4.277) and memory (p < 0.01; t = 2.801) [47]. This study has shown that engaging in meditation and yoga enhances working memory, relaxes an individuals’ breathing, and reduces anxiety [47]. Making lifestyle modifications such as incorporating physical exercise in the form of strenuous exercise in the gym or peaceful meditation, which is dependent on personal preferences, can significantly enhance a medical students’ experience and may even lead to reduced reliance and consumption of stimulatory substances.

Limitations of the study

Some limitations of this paper are that we focused on a small population of highly stressed individuals when considering the use of stimulants. Due to this limitation, the studies discussed throughout this paper and the effects of stimulant use may not be generalizable to the majority of the population, which may use stimulants for other purposes. However, this review may be relevant to students and professionals pursuing careers in various fields of medicine that require extensive educational training and long work hours. Further research is indicated to assess the effects of long-term use of stimulants on behavioral changes among medical students. Studies evaluating chronic use of psychostimulants and their effects of neuronal damage or changes in IQ levels are also indicated as the use of energy drinks is relatively new and the effects of chronic use are not well established. Longitudinal studies and the use of stimulants in conjunction with non-pharmacological supplements may provide more direction in cognitive improvement and increased alertness while abstaining from excessive consumption of stimulatory substances.

## Conclusions

The use of stimulants, be that coffee, energy drinks, or amphetamines, often entices students with the idea that they can greatly enhance their cognitive ability within a short period of time and with minimal effort. This idea is largely unfounded. While stimulants may provide certain benefits when taken in small doses and moderation, they quickly become detrimental with larger doses and increased frequency and may lead to reliance. Thus, students should be encouraged to seek alternative methods of decreasing stress and improving productivity. The methods suggested are not quite as simple as taking a pill or drinking a caffeinated beverage, however, when used properly they are more sustainable and contribute to a greater level of overall health. In order to foster sustainable and productive habits, students should be encouraged to engage in activities that support cognitive performance without the use of stimulants, such as exercise, meditation, and a healthy diet. Medical schools should also be encouraged to support students by making resources available to them, such as the best academic resources for learning, active learning involving the students, and other healthy recreations like gyms, yoga classes, and targeted information on stress relief. Though it appears beneficial to restrict the use of stimulants from the diet entirely, in low doses and in combination with other healthy behaviors, stimulant use may be tolerated within healthy individuals.

## References

[1] Fond G, Gavaret M, Vidal C, Brunel L, Riveline JP, Micoulaud-Franchi JA, Domenech P (2016). (Mis)use of prescribed stimulants in the medical student community: motives and behaviors: a population-based cross-sectional study. Medicine (Baltimore).

[2] Arria AM, Caldeira KM, Vincent KB (2017). Do college students improve their grades by using prescription stimulants nonmedically?. Addict Behav.

[3] Patrick ME, Griffin J, Huntley ED, Maggs JL (2018). Energy drinks and binge drinking predict college students' sleep quantity, quality, and tiredness. Behav Sleep Med.

[4] Liberati A, Altman DG, Tetzlaff J (2009). The PRISMA statement for reporting systematic reviews and meta-analyses of studies that evaluate healthcare interventions: explanation and elaboration. BMJ.

[5] Miranda M, Barbosa M (2021). Use of cognitive enhancers by Portuguese medical students: do academic challenges matter?. Acta Med Port.

[6] Alrakaf FA, Binyousef FH, Altammami AF, Alharbi AA, Shadid A, Alrahili N (2020). Illicit stimulant use among medical students in Riyadh, Saudi Arabia. Cureus.

[7] Acosta DL, Fair CN, Gonzalez CM (2019). Nonmedical use of d-amphetamines and methylphenidate in medical students. P R Health Sci J.

[8] Haas GM, Momo AC, Dias TM, Ayodele TA, Schwarzbold ML (2019). Sociodemographic, psychiatric, and personality correlates of non-prescribed use of amphetamine medications for academic performance among medical students. Braz J Psychiatry.

[9] De Bruyn S, Wouters E, Ponnet K, Van Hal G (2019). Popping smart pills in medical school: are competition and stress associated with the misuse of prescription stimulants among students?. Subst Use Misuse.

[10] Fallah G, Moudi S, Hamidia A, Bijani A (2018). Stimulant use in medical students and residents requires more careful attention. Caspian J Intern Med.

[11] Retief M, Verster C (2016). Prevalence and correlates of non-medical stimulants and related drug use in a sample of South African undergraduate medical students. S Afr J Psychiatr.

[12] Wasserman JA, Fitzgerald JE, Sunny MA, Cole M, Suminski RR, Dougherty JJ (2014). Nonmedical use of stimulants among medical students. J Am Osteopath Assoc.

[13] Kudlow PA, Naylor KT, Xie B, McIntyre RS (2013). Cognitive enhancement in Canadian medical students. J Psychoactive Drugs.

[14] Emanuel RM, Frellsen SL, Kashima KJ, Sanguino SM, Sierles FS, Lazarus CJ (2013). Cognitive enhancement drug use among future physicians: findings from a multi-institutional census of medical students. J Gen Intern Med.

[15] Habibzadeh A, Alizadeh M, Malek A, Maghbooli L, Shoja MM, Ghabili K (2011). Illicit methylphenidate use among Iranian medical students: prevalence and knowledge. Drug Des Devel Ther.

[16] Tuttle JP, Scheurich NE, Ranseen J (2010). Prevalence of ADHD diagnosis and nonmedical prescription stimulant use in medical students. Acad Psychiatry.

[17] Samaha A, Al Tassi A, Yahfoufi N, Gebbawi M, Rached M, Fawaz MA (2020). Data on the relationship between caffeine addiction and stress among Lebanese medical students in Lebanon. Data Brief.

[18] Aslam HM, Mughal A, Edhi MM (2013). Assessment of pattern for consumption and awareness regarding energy drinks among medical students. Arch Public Health.

[19] Hidiroglu S, Tanriover O, Unaldi S, Sulun S, Karavus M (2013). A survey of energy-drink consumption among medical students. J Pak Med Assoc.

[20] Ríos JL, Betancourt J, Pagán I (2013). Caffeinated-beverage consumption and its association with socio-demographic characteristics and self-perceived academic stress in first and second year students at the University of Puerto Rico Medical Sciences Campus (UPR-MSC). P R Health Sci J.

[21] Casuccio A, Bonanno V, Catalano R, Cracchiolo M, Giugno S, Sciuto V, Immordino P (2015). Knowledge, attitudes, and practices on energy drink consumption and side effects in a cohort of medical students. J Addict Dis.

[22] Usman A, Bhombal ST, Jawaid A, Zaki S (2015). Energy drinks consumption practices among medical students of a private sector university of Karachi, Pakistan. J Pak Med Assoc.

[23] Weyandt LL, Oster DR, Marraccini ME, Gudmundsdottir BG, Munro BA, Rathkey ES, McCallum A (2016). Prescription stimulant medication misuse: where are we and where do we go from here?. Exp Clin Psychopharmacol.

[24] Arria AM, Geisner IM, Cimini MD (2018). Perceived academic benefit is associated with nonmedical prescription stimulant use among college students. Addict Behav.

[25] Jain R, Chang CC, Koto M, Geldenhuys A, Nichol R, Joubert G (2017). Non-medical use of methylphenidate among medical students of the University of the Free State. S Afr J Psychiatr.

[26] Husain M, Mehta MA (2011). Cognitive enhancement by drugs in health and disease. Trends Cogn Sci.

[27] Vasan S, Olango GJ (2021). Amphetamine Toxicity.

[28] Faraone SV (2018). The pharmacology of amphetamine and methylphenidate: relevance to the neurobiology of attention-deficit/hyperactivity disorder and other psychiatric comorbidities. Neurosci Biobehav Rev.

[29] Weyandt LL, White TL, Gudmundsdottir BG, Nitenson AZ, Rathkey ES, De Leon KA, Bjorn SA (2018). Neurocognitive, autonomic, and mood effects of Adderall: a pilot study of Healthy College Students. Pharmacy (Basel).

[30] Miller GM (2011). The emerging role of trace amine-associated receptor 1 in the functional regulation of monoamine transporters and dopaminergic activity. J Neurochem.

[31] Rotenstein LS, Ramos MA, Torre M (2016). Prevalence of depression, depressive symptoms, and suicidal ideation among medical students: a systematic review and meta-analysis. JAMA.

[32] De Sanctis V, Soliman N, Soliman AT, Elsedfy H, Di Maio S, El Kholy M, Fiscina B (2017). Caffeinated energy drink consumption among adolescents and potential health consequences associated with their use: a significant public health hazard. Acta Biomed.

[33] Cabezas-Bou E, De León-Arbucias J, Matos-Vergara N (2016). A survey of energy drink consumption patterns among college students at a mostly hispanic university. J Caffeine Res.

[34] Devi SSL, Abilash SC, Basalingappa S (2018). The rationale of caffeine consumption and its symptoms during preparatory and non-preparatory days: a study among medical students. Biomed Pharmacol J.

[35] Willson C (2018). The clinical toxicology of caffeine: a review and case study. Toxicol Rep.

[36] Chen JQA, Scheltens P, Groot C, Ossenkoppele R (2020). Associations between caffeine consumption, cognitive decline, and dementia: a systematic review. J Alzheimers Dis.

[37] Sherman SM, Buckley TP, Baena E, Ryan L (2016). Caffeine enhances memory performance in young adults during their non-optimal time of day. Front Psychol.

[38] Evans J, Richards JR, Battisti AS (2020). Caffeine. https://www.ncbi.nlm.nih.gov/books/NBK519490/.

[39] Konishi Y, Hori H, Ide K (2018). Effect of single caffeine intake on neuropsychological functions in healthy volunteers: a double-blind placebo-controlled study. PLoS One.

[40] Sorkin BC, Camp KM, Haggans CJ (2014). Executive summary of NIH workshop on the use and biology of energy drinks: current knowledge and critical gaps. Nutr Rev.

[41] Bernard BN, Louise LC, Louise D (2018). The effects of carbohydrates, in isolation and combined with caffeine, on cognitive performance and mood-current evidence and future directions. Nutrients.

[42] Hashem KM, He FJ, MacGregor GA (2017). Cross-sectional surveys of the amount of sugar, energy and caffeine in sugar-sweetened drinks marketed and consumed as energy drinks in the UK between 2015 and 2017: monitoring reformulation progress. BMJ Open.

[43] Fletcher EA, Lacey CS, Aaron M, Kolasa M, Occiano A, Shah SA (2017). Randomized controlled trial of high-volume energy drink versus caffeine consumption on ECG and hemodynamic parameters. J Am Heart Assoc.

[44] Zarobkiewicz MK, Woźniakowski MM, Wawryk-Gawda E, Sławiński MA, Halczuk P, Korolczuk A, Jodłowska-Jędrych B (2018). Decrease in lipid droplets in adrenal cortex of male Wistar rats after chronic exposure to energy drinks. Medicina (Kaunas).

[45] Curran CP, Marczinski CA (2017). Taurine, caffeine, and energy drinks: reviewing the risks to the adolescent brain. Birth Defects Res.

[46] Kennedy S (2018). Raising awareness about prescription and stimulant abuse in college students through on-campus community involvement projects. J Undergrad Neurosci Educ.

[47] Joice PPS, Manik KA, Sudhir PK (2018). Role of yoga in attention, concentration, and memory of medical students. Natl J Physiol Pharm Pharmacol.

